# A Rare Odontogenic Cyst: Gingival Cyst of the Adult. A Case Report and Review of the Literature in Elderly Patients

**DOI:** 10.1155/2023/4827611

**Published:** 2023-07-26

**Authors:** Martina Frigerio, Rami Al Eid, Tommaso Lombardi

**Affiliations:** ^1^Division of Gerodontology and Removable Prosthodontics, University Clinics of Dental Medicine, University of Geneva, Geneva, Switzerland; ^2^Undergraduate Clinics, University Clinics of Dental Medicine, University of Geneva, Geneva, Switzerland; ^3^Laboratory of Oral and Maxillofacial Pathology, Unit of Oral Medicine and Oral Maxillofacial Pathology, University Hospitals of Geneva and Faculty of Medicine, University of Geneva, Geneva, Switzerland

## Abstract

Gingival cysts of adults (GCAs) are a relatively rare type of odontogenic cysts developing from the remnants of the dental lamina. Although GCAs generally affect individuals below the age of 65, their findings in older patients may exceptionally occur. Usually asymptomatic, they present as small, generally solitary nodules, located on the attached gingiva, primarily in the mandibular canine and premolar regions. This report highlights a rare case of a solitary GCA in an 82-year-old patient, who presented with a discrete nodule located between the right mandibular canine and first premolar. An excisional biopsy was performed, and the diagnosis of GCA was confirmed by the histological examination. No recurrence was observed during the 18-month follow-up period. Additionally, a literature review was conducted to analyse the clinical and pathological characteristics of GCAs in patients aged 65 and older. By providing details of this rare entity, our case study highlights the importance of considering GCAs when diagnosing gingival lesions in geriatric patients. By increasing our understanding of GCAs in the elderly population, our findings can help improve diagnosis as well as management strategies for these lesions.

## 1. Introduction

Gingival cysts of adults (GCAs) are slow-growing, odontogenic, developmental cysts originating from the rests of dental lamina [1]. GCAs are considered rare lesions, accounting for approximately 0.3% of all odontogenic cysts [2, 3]. Most studies agree that the mandible is the most common site for GCAs, mainly located in the vestibular attached gingiva of the canine and first premolar areas. Patients describe these lesions as slow-growing, painless swellings. Clinically, GCAs manifest as small, solitary nodules, or vesicles, usually not exceeding 6 mm in size [4, 5]. Although cases of multiple lesions have been reported, they are rare [6]. The color of the lesion is generally flesh-colored, occasionally displaying a bluish hue due to the presence of cystic fluid [5, 7]. In terms of patient demographics, GCAs have a slight female predilection [3, 7–10]. The age at diagnosis varies with a peak incidence in the fifth and sixth decades of life [2, 3, 5–9]. Cases affecting patients older than 65 years of age are rare (Table 1).

Panoramic and intraoral radiographic examinations usually do not reveal bone involvement, unless significant bone resorption has occurred due to cystic pressure [4, 8, 11].

Microscopically, GCAs are characterized by an uninflamed, connective tissue wall, lined with a thin, squamous, or cuboidal epithelium. Focal areas of epithelial thickening, appearing as plaques, may be present, sometimes containing glycogen-rich clear cells [4, 5, 7].

The treatment of choice for GCAs is surgical excision, with a favorable prognosis and extremely rare recurrence [5].

This article describes the case of an 82-year-old presenting with GCA, with the aim of providing a comprehensive overview of the clinical, radiological, and histological findings. The primary objective is to assist practitioners in recognising and effectively managing this particular pathology. Furthermore, a literature review focusing on GCAs in the elderly population has been included to provide a comprehensive overview.

## 2. Case Presentation

An 82-year-old female patient presented to the dental office for a routine checkup in August 2020, reporting the presence of a slow-growing, painless swelling on the lower right gingiva, which she had noticed several months prior. The patient did not recall any history of trauma in that area. She had previously undergone some dental therapy at the nursing home but no recent treatment. She had a poor dental status, including tooth wearing, discolorations, old resin fillings, and multiple missing teeth, which had not been replaced. Despite no periodontal problems detected, a slight loss of attachment was found. Her medical history included colorectal adenocarcinoma resection 5 years earlier, which prompted her to seek dental advice in order to investigate the exact nature of the gingival swelling. Her medical history also included non-insulin dependent type II diabetes, hypercholesterolemia, and hypertension, all of which were untreated. The patient reported no history of smoking or other tobacco use.

**Figure 1:**
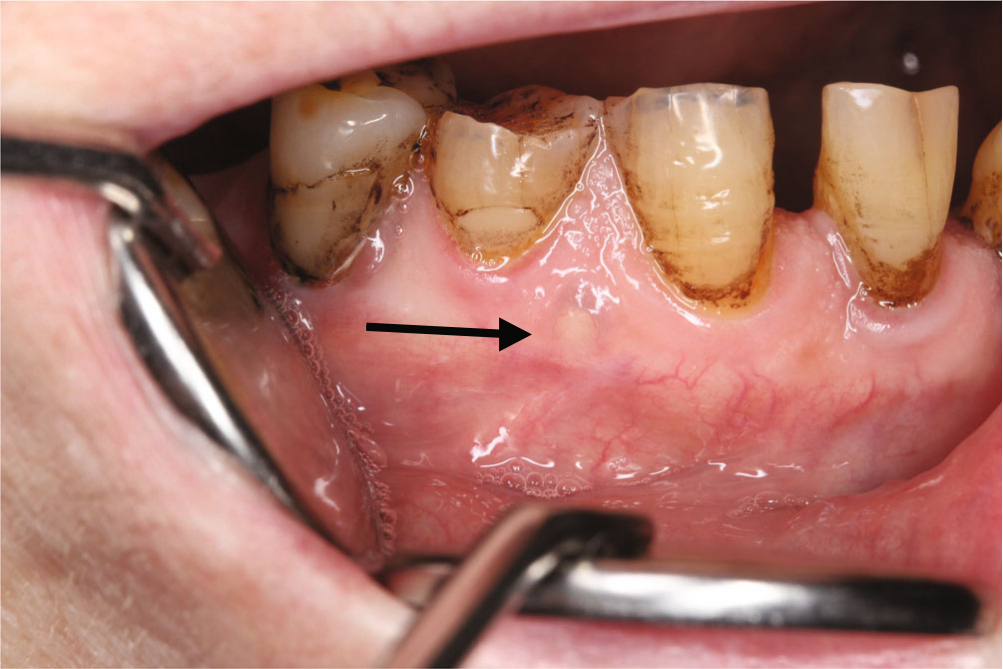
Clinical view at initial examination.

**Figure 2:**
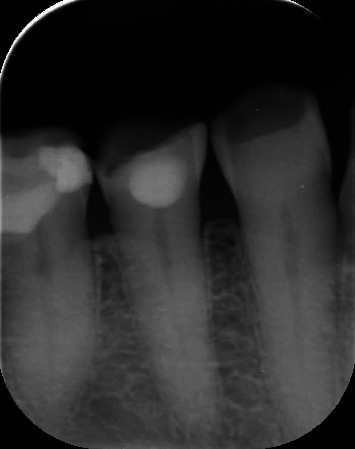
Intraoral periapical radiograph revealing no bone involvement and showing signs of tooth wear.

On clinical examination, a small, solitary, translucent, yellowish nodule measuring 3 × 3 mm with a smooth round surface was found. The lesion was located on the attached gingiva below the interdental papilla, between the lower right mandibular canine (tooth #43) and first premolar (tooth #44). It appeared firm and asymptomatic with a sessile base (Figure 1). Pulp testing of the lower right canine and premolars indicated normal pulp vitality. Radiographically, no findings were suggesting osseous involvement (Figure 2). Based on the clinical and radiographic findings, a provisional diagnosis of a cystic gingival lesion was made. The differential diagnosis included other small oral nodular lesions such as a fibroma, a retained foreign body, a lipoma, and, although rare, peripheral ameloblastoma and peripheral odontogenic keratocyst. The diagnosis of lateral periodontal cyst (LPC) was excluded due to the absence of radiological findings.

Because of the size of the lesion, an excisional biopsy was performed under local anesthesia (Septanest 4% with adrenaline 1 : 200.000, SEPTODONT, 58 rue du Pont de Créteil, 94107 Saint-Maur-des-Fossés, France). Injections were made at a distance of approximately 3 mm from the lesion in order not to damage and therefore impinge on the lesion tissue and to ensure good microscopic analysis. Two injection points were chosen, the first above the lesion, at the level of the papilla, and the second below, in the vestibular region. Approximately, 0.5 ml of solution was administered. A spindle-shaped incision was made with a blade scalpel (blade n° 15) extending approximately 1 mm beyond the lesion edge in healthy tissue. The overlying mucosa and the underlying connective tissue were removed, and a translucent, yellowish liquid discharge was observed during the procedure. The excised specimen was fixed in 10% buffered formalin solution and submitted for histopathological examination. Two sutures were placed for wound closure (Vicryl Rapide^™^ 4-0, Ethicon^®^ Inc., Somerville, NJ, USA).

**Figure 3:**
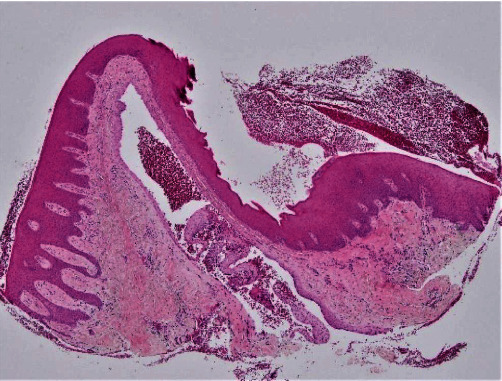
Mucosal fragment lined by stratified squamous epithelium supported by fibrous connective tissue containing a cystic space (H&E, ×4).

**Figure 4:**
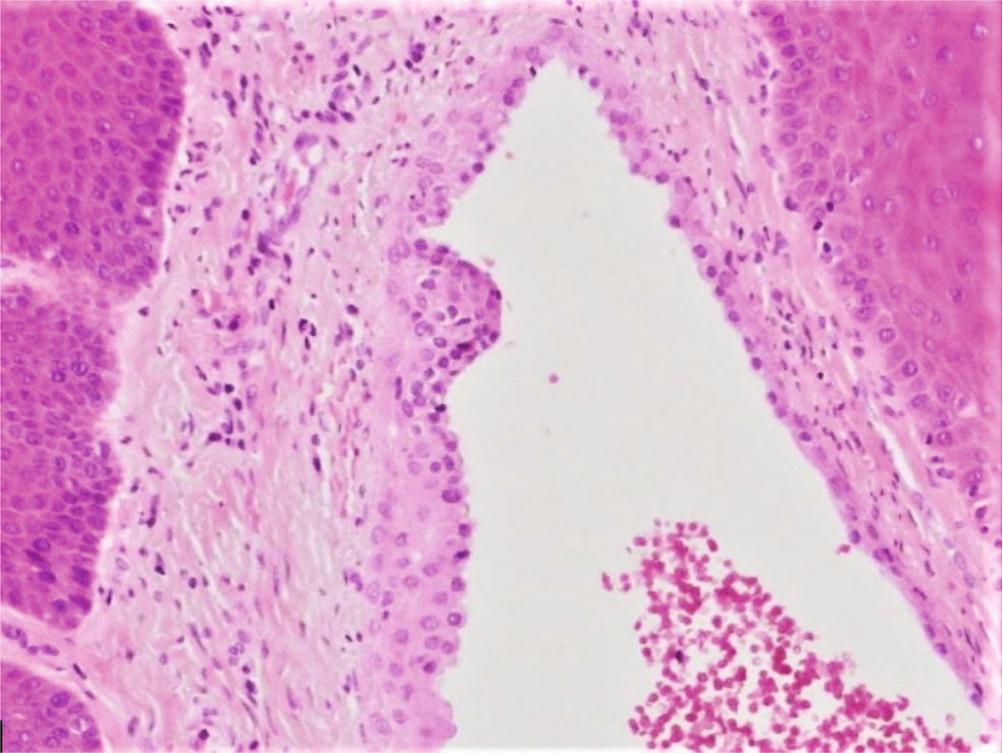
The cystic lumen is lined by 2–5 cell thick squamous and cuboidal cells (H&E, ×20).

**Figure 5:**
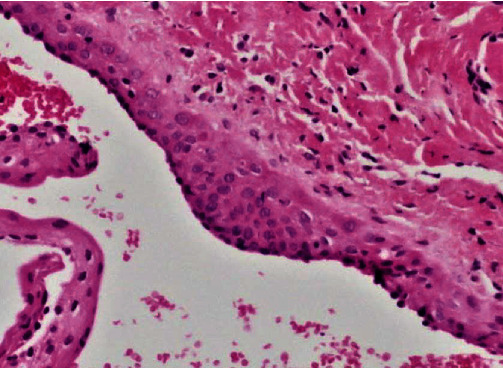
High-power view showing an epithelial plaque (H&E, ×40).

Macroscopic inspection of the excised tissue revealed a grayish nodular fragment of soft tissue, measuring approximately 4 × 3 × 2 mm. Histological analysis of the gingival tissue sections stained with haematoxylin and eosin solution revealed a cystic cavity lined with two or three layers of cuboidal cells of non-keratinized stratified squamous epithelium (Figures 3 and 4). Focal epithelial plaques containing clear cells were also observed (Figure 5). The fibrous wall appeared uninflamed. The histopathological diagnosis confirmed a GCA.

The sutures were removed after 2 weeks, and the patient reported no postoperative complications. Although sutures are usually removed 7–10 days after the intervention, we decided to wait until the patient's next scheduled appointment for other dental treatments, having taken into account her age and in order to minimize the number of appointments.

**Figure 6:**
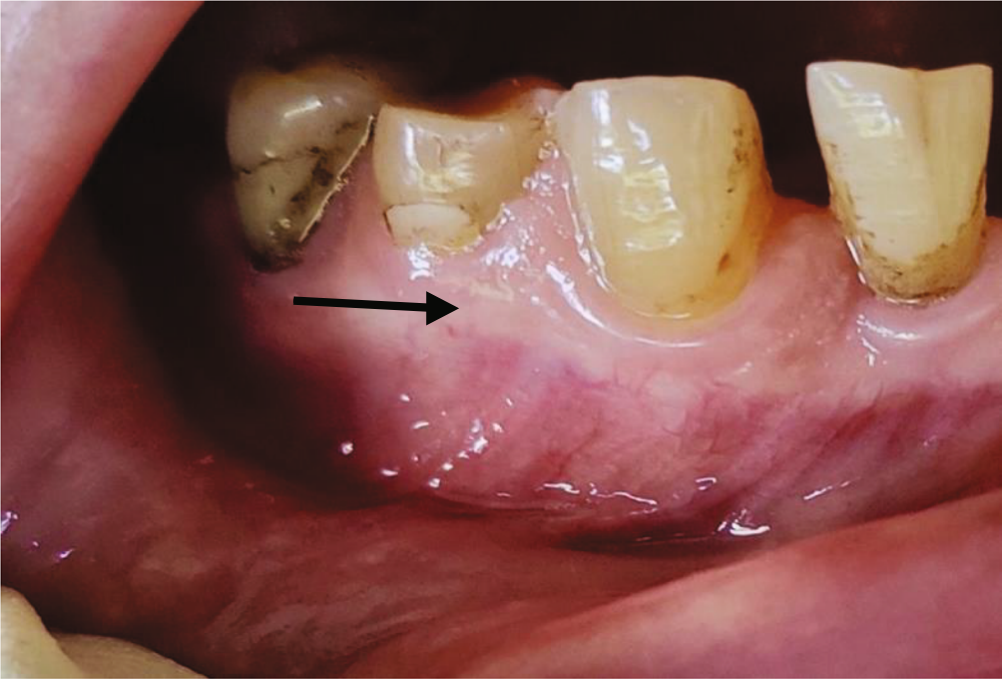
Clinical view at 2.5-year follow-up examination.

Regular follow-up appointments have shown no signs of recurrence for over 2.5 years (Figure 6).

## 3. Discussion

GCAs are rare developmental lesions, with about 170 cases described in the literature over the past 70 years worldwide [2, 8, 12, 13]. Unfortunately, in most studies, they are only cited among odontogenic cysts or as a subgroup without providing a detailed description of each case's characteristics [6, 10, 11, 14]. Furthermore, in the past, GCAs may have been misdiagnosed for LPCs [11, 14–16]. Indeed, GCAs and LPCs share many clinical and histopathological features, which suggest that they share common histogenesis and represent the intraosseous and extraosseous manifestations of the same lesion [11, 17]. We, therefore, selected only case reports from 1970 onwards in our literature review. Within this period, we identified 12 studies reporting 15 cases of GCAs in patients over 65 years of age. The demographic and clinical characteristics of each case are detailed in Table 1.

In the 15 reported cases of GCAs, the patients' age ranged from 68 to 80 years. Our case represents, to the best of our knowledge, the eldest reported patient with a GCA, the patient being 82 years old.

The majority of the cases occurred in the mandibular region and were located on the attached gingiva in the lateral incisor–canine–premolar area. The size of the lesion was reported in 10 cases, normally being less than 5 mm. Only two reports described larger lesions, measuring 1.5 and 2 cm, respectively [2, 4]. Eight cases were observed in female patients, and four cases were observed in male patients. Unfortunately, information about gender is missing for three cases. As in our study, the lesions presented as non-painful, firm, nodular swellings, with a pale pink, grayish, or yellowish color.

In our case, there was no radiographic evidence of bone involvement, and it seems that most other studies share this finding, although information is lacking for five cases. Another case showed radiographic radiolucency [18], which may be explained by pressure-induced resorption of the adjacent bone caused by the lesion.

Based on the clinical presentation, the differential diagnosis of GCA included other lesions presenting as gingival swellings, such as a fibroma or a retained foreign body. However, our patient had no history of trauma in the region. A lipoma may also present with the same aspect of the above described lesion, although it is extremely rare in the attached gingiva, and the histopathological diagnosis is unequivocal [19]. Although very rare, peripheral ameloblastoma and peripheral odontogenic keratocyst can also exhibit the same clinical characteristics [20–22]. As previously mentioned, LPC is another important lesion to be considered in the differential diagnosis. LPCs share the same histopathological appearance of GCAs. They are also believed to develop from the rests of dental lamina, but they expand from the periodontium, while gingival cysts arise in the gingiva without communication with the periodontal ligament [14]. The radiographic and surgical findings can help in establishing a correct differential diagnosis [8].

GCAs are treated by simple excision with minimal margin involvement and submitted for histopathological examination to establish the diagnosis, with usually no tendency to recur [13].

In conclusion, GCAs are considered uncommon, slow-growing, benign, developmental cysts of odontogenic origin, typically located in the mandibular attached gingiva in the canine–premolar area. Although they are rare in the elderly, they should be included in the differential diagnosis of gingival swellings.

## Figures and Tables

**Table 1:** Published case reports and literature reviews on gingival cysts of the adult in patients over 65 years of age since 1970.

Reference	*N*	Age (years)	Gender (M : F)	Max/Mand	Region	Size (mm)	Radiographic evidence	Bone involvement
Young et al. [16]	1	68	F	Mand	Canine	3 mm	NR	No
Buchner and Hansen [14]	1	77	NR	NR	NR	NR	NR	NR
Wysocki et al. [11]	1	75	NR	NR	NR	NR	NR	NR
Gregg and O'Brien [17]	1	68	M	Max	Canine	NR	None	NR
Giunta [6]	1	80	NR	NR	NR	NR	None	NR
Cunha et al. [18]	1	69	F	Mand	Lateral incisor–canine	5 mm	Yes	Yes
Sato et al. [4]	1	78	M	Mand	Lateral incisor	20 mm	None	Yes
Ojha et al. [23]	1	76	F	Mand	NR	5 mm	None	NR
Wagner et al. [8]	1	75	F	Mand	Central incisor–lateral incisor	5 mm	None	Yes
Brooks et al. [12]	1	68	F	Mand	Canine–first premolar	NR	None	No
Karmakar et al. [13]	1	76	M	Mand	Lateral incisor–canine	5 mm	None	No
Viveiros et al. [2]	1	78	F	Mand	Canine/first premolar	4 mm	None	NR
	2	78	F	Mand	Lateral incisor–canine	2 mm	None	NR
	3	76	M	Mand	First premolar	15 mm	NR	NR
	4	79	F	Mand	Premolar	5 mm	None	No
This study (2022)	1	82	F	Mand	Canine/first premolar	3 mm	None	No

*N*, case number; M : F, male : female; Max : Mand, maxilla : mandible; NR, not reported.

## Data Availability

We confirm that all relevant data supporting the conclusions of this case report are included within the article. The data used to write and illustrate this case report, including patient information, clinical records, and laboratory results, are protected by patient privacy regulations and are not publicly available. If requested, however, and after obtaining the necessary ethical and institutional permissions, the anonymised data may be made available to qualified researchers for research purposes. Requests for access to data should be addressed to Frigerio Martina at https://martina.frigerio@unige.ch.
